# Electron paramagnetic resonance spectroscopy for analysis of free radicals in zebrafish

**DOI:** 10.1371/journal.pone.0318212

**Published:** 2025-02-21

**Authors:** Mitra Sabetghadam Moghadam, Eli Wiens, Sébastien Gauvrit, Ramaswami Sammynaiken, Michelle M Collins

**Affiliations:** 1 Department of Anatomy, Physiology, and Pharmacology, College of Medicine, University of Saskatchewan, Saskatoon, Saskatchewan, Canada; 2 Saskatchewan Structural Sciences Centre, University of Saskatchewan, Saskatoon, Saskatchewan, Canada; Indian Institute of Technology Guwahati, INDIA

## Abstract

Electron paramagnetic resonance (EPR) is an excellent choice for detecting free radicals in biological samples. Biologically relevant radicals are extremely short-lived and cannot be detected directly, emphasizing the need for an appropriate compound to generate stable adducts that can be measured by EPR. Spin trapping with nitrone compounds like 5,5-dimethyl-1-pyrroline N-oxide (DMPO) is a method commonly employed for detecting free radicals. However, due to the instability of nitrone radical adducts, using the cell-permeable 1-hydroxy-3-methoxycarbonyl-2,2,5,5-tetramethyl pyrrolidine (CMH) appears to be a more effective approach within biological tissues. Here, we compare the use of DMPO and CMH to detect the most abundant reactive oxygen species radical, superoxide ($O_{2}^{\cdot -}$), in zebrafish and present an optimized protocol for performing EPR with a CMH spin probe in both zebrafish hearts and larvae. Together, our data suggest that EPR using the CMH probe is a reliable method to detect $O_{2}^{\cdot -}$ in zebrafish pathologies linked to oxidative stress, such as cardiovascular diseases.

## Introduction

Oxidative stress arises from an imbalance between the formation of free radicals and the mechanisms responsible for their removal. Free radicals are identified by the presence of unpaired electrons in atoms, molecules, or ions [1]. This characteristic makes them highly reactive in biological systems. Reactive oxygen species (ROS) are produced by transferring electrons to oxygen, mainly in the mitochondrial electron transport chain [2]. ROS may also originate as byproducts of intracellular enzymes, like nicotinamide adenine dinucleotide phosphate (NADPH) oxidases [3], xanthine oxidases [4], and uncoupled nitric oxide synthase [5]. Elevated ROS levels or impairment of the antioxidant defense system have been linked to a range of pathological conditions, including cancer [6], inflammatory disorders [7], and cardiovascular disease [8]. Therefore, scientific interest is keenly directed toward understanding ROS levels and their association with diseases.

Zebrafish have emerged as a powerful model to study cellular redox and how ROS influences disease *in vivo*. Zebrafish are well-suited to study mechanisms and effects of oxidative stress in response to different environmental and pathological factors that induce excessive ROS production [9,10]. The small size and optical transparency of embryonic and larval zebrafish make them highly amenable to high-resolution imaging of live animals [11]. Not only are zebrafish used to study disease pathogenesis, including cardiovascular disorders [12,13] they are also a popular model for toxicological and drug discovery studies [14]. A notable advantage in drug discovery studies is the straightforward application of chemicals at embryonic and juvenile stages. This is especially beneficial for water-soluble compounds, facilitating the evaluation of compound effects on redox balance [15]. Additionally, zebrafish share significant genetic and physiological similarities with humans, making it a relevant and practical model for investigating various aspects of oxidative stress related to cardiovascular diseases [16], including exploring the impact of ROS on cardiac function, elucidating the role of antioxidant defense mechanisms, and unraveling the molecular pathways involved in oxidative stress-induced damage [8]. However, our knowledge about oxidative stress in the context of the whole animal, in specific tissues, and various compartments of a cell is still limited [17,18]. Therefore, developing and improving techniques to measure ROS in zebrafish is critical.

Several methods are available for detecting ROS in zebrafish, including chemical probes and genetically encoded biosensor lines [11]. Commonly used chemical and engineered redox probes include 2’,7’-dichlorodihydrofluorescein diacetate (H2DCFDA) [19], dihydroethidium (DHE), and mitochondrial-targeted DHE MitoSOX Red [20]. These probes undergo oxidation by ROS, leading to modifications in their chemical structure and generating fluorescence. However, most commercially available ROS detection probes may lack precise qualitative and quantitative analysis of oxidative stress levels in living cells [11,21]. Genetically engineered redox-sensitive fluorescent proteins serve as biosensors with enhanced specificity, enabling real-time monitoring of endogenous ROS levels in transgenic zebrafish [11,22]. Some genetically-encoded biosensor probes that contribute to *in vivo* redox studies include HyPer (a specific H_2_O_2_-sensitive fluorescent probe) [23], redox-sensitive green fluorescent protein (roGFP) [8,24], redox-sensitive yellow fluorescent protein (rxYFP) [11], and circularly permuted yellow fluorescent protein (cpYFP) [25]. Both approaches have distinct advantages and disadvantages. Chemical probes may have low sensitivity for various oxidants, while biosensor lines can detect ROS with more sensitivity and selectivity, providing subcellular resolution [10]. However, generating biosensor lines can be labour intensive, requiring time to establish a stable transgene and access to appropriate imaging tools. Further, *in vivo* live imaging of adult zebrafish is challenging [26].

Electron paramagnetic resonance (EPR), also known as electron spin resonance (ESR), is a powerful method for measuring oxidative stress. EPR is a spectroscopy-based method to directly detect materials containing unpaired electrons. EPR involves the detection of electron spin excitation within an applied magnetic field [32]. However, free radicals such as hydroxyl ( ⋅ *OH*) and $O_{2}^{\cdot -}$ exhibit distinct behavior in biological systems. Biological relevant radicals have a short lifetime in the nanosecond ranges, depending on the reactivity and presence of cellular antioxidants. Within *in vivo* enzymatic systems such as cells, the free radical concentration usually remains below 1 nanomolar [33], falling below the detection limit of EPR spectroscopy ($10^{-7}\;-\;10^{-8}$ M) [34]. Hence, direct detection of radicals in biological systems using EPR becomes impossible. Instead, chemical compounds are needed to convert these highly unstable radicals into more stable forms. This process allows a nanomolar radical concentration to accumulate into high micromolar concentrations called spin adducts or radical adducts, which can then be detected by EPR.

There are two key approaches in EPR spectroscopy: using spin traps and spin probes [35]. EPR can be performed using a chemical spin trap that reacts with biological samples containing unpaired electrons to create a spin adduct. Spin traps produce specific spin adducts depending on the type of original free radical, resulting in specific spectra [36]. 5,5-dimethyl-1-pyrroline-N-oxide (DMPO) is a widely used nitrone spin trap capable of reacting with distinct free radicals, such as $O_{2}^{\cdot -}$ and  ⋅ *OH*, generating DMPO/ ⋅ *OOH* and DMPO/ ⋅ *OH* adducts, respectively; each spin adduct is then captured using the EPR spectrometer as a specific spectrum, though the rapid conversion of DMPO/ ⋅ *OOH* to DMPO/ ⋅ *OH* limits the ability to observe the former [37]. The use of spin probes for detecting ROS in biological samples has been widely employed. One class of compounds, the cyclic hydroxylamines, has proven extremely effective for use in tissues and cultured cells. Probes do not react with free radicals to form a covalent bond. Instead, probes are oxidized by free radicals present in the system, forming an oxidized form of the probe, nitroxide, with a half-life of several hours which is detectable by EPR [38]. The cyclic hydroxylamine probe,1-hydroxy-3-methoxycarbonyl-2,2,5,5-tetramethylpyrrolidine (CMH), is oxidized and produces CM ⋅  nitroxide in the presence of $O_{2}^{\cdot -}$ [35].

EPR has been used for detecting free radicals such as ROS in various biological samples, including frozen biopsies [27], blood [28], and animal models including zebrafish embryos [29], mice [30], and pigs [31]. Despite these applications, the use of EPR in zebrafish, particularly in adult or juvenile tissues, remains underexplored. Here, we compare the use of DMPO spin trap and CMH spin probe and present an optimized EPR-based method to measure the most abundant ROS, superoxide ($O_{2}^{\cdot -}$) in whole larvae and isolated hearts from juvenile and adult zebrafish.

## Materials and methods

**Fig 1:**
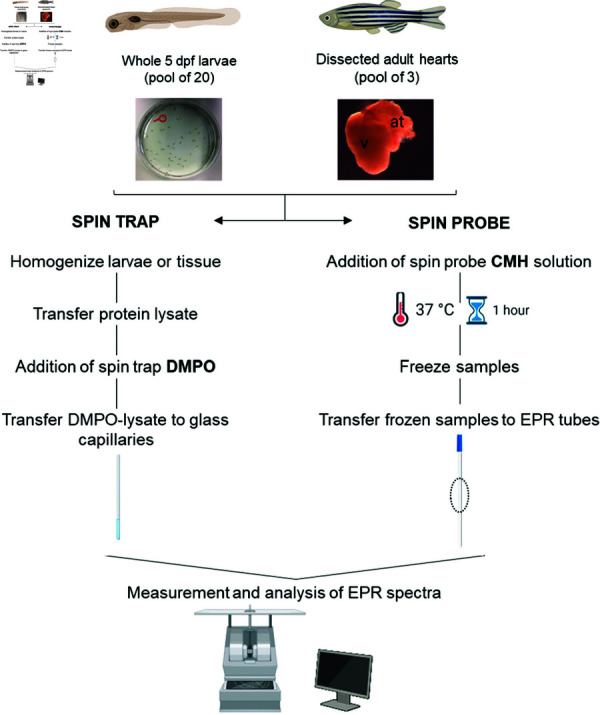
Schematic overview of the protocol showing sample preparation from either pools of larval zebrafish or dissected adult cardiac tissue. Samples are processed using the DMPO spin trap or CMH spin probe by EPR spectroscopy. Figure icons created in BioRender.

The protocol (Fig 1) described in this peer-reviewed article is published on protocols.io (https://dx.doi.org/10.17504/protocols.io.q26g7mdeqgwz/v1) and is included for printing purposes as S1 File.

## Results

**Fig 2:**
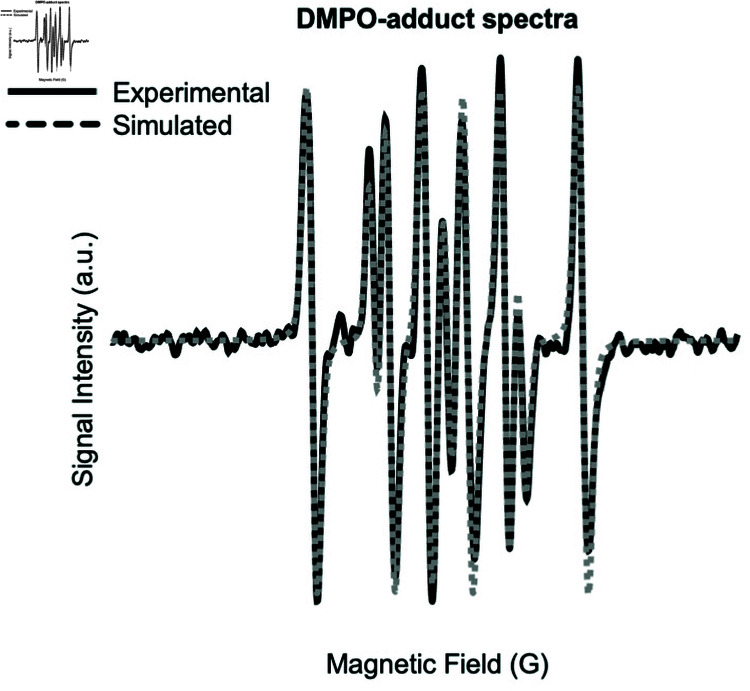
Representative experimental and simulated EPR spectra of DMPO (100 mM) in a pool/20 of zebrafish larvae. The dashed line represents the experimental EPR spectrum, and the solid line shows the simulated spectrum.

We compared the suitability of the DMPO spin trap and CMH spin probe for detecting radicals in zebrafish samples using EPR. Various DMPO concentrations (10 mM, 50 mM, and 100 mM) were used to determine the optimal concentration for our biological samples, and 100 mM was selected to obtain a sufficiently large signal. However, the DMPO/ ⋅ *OH* signal was generally weak relative to interference from other radicals. To identify other radical species spectral subtraction was used to isolate individual components of the signal, which were then simulated. The simulated radicals (Table 1), were then used to fit the experimental data (Fig 2). The hyperfine couplings of radical 1, are similar to carbon centered adducts (e.g. alkyl), but also similar to hydroxyalkyl [39]. Radical 2 is consistent with an aminoxyl impurity derived from DMPO itself, and in general a similar combination of radicals is observed in degraded DMPO [40]. A similar combination of radicals with slightly different hyperfine couplings was also reported in reperfused rabbit heart, and by using superoxide dismutase were determined to be mainly formed by reaction with $O_{2}^{\cdot -}$ [41]. Differences between the buffer composition used in this study with others prevent direct comparison of hyperfine constants.

**Table 1:** Fitted parameters for DMPO radical adducts in zebrafish larval spectrum. Provided g-factor and hyperfine splitting (HFS) for each species in the simulated spectrum.

	g-factor	aN (G)	aH (G)
Radical 1	2.006	15.5	23.0
Radical 2	2.006	14.7	–
DMPO/ ⋅ *OH*	2.006	14.9	14.9

**Fig 3:**
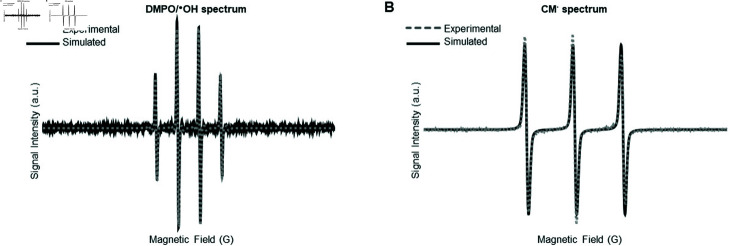
Stable radical adducts generated from the DMPO spin trap from a Fenton reaction and CMH spin probe reacting with $O_{2}^{\cdot -}$. (A) Representative experimental and simulated EPR spectra of DMPO (100 mM) in the Fenton reaction system. The dashed line represents an experimental EPR spectrum, while the solid line shows a simulated spectrum. (B) Representative experimental and simulated EPR spectra of CMH (1 mM) in zebrafish adult hearts. The dashed line represents an experimental EPR spectrum, while the solid line shows the simulated spectrum.

To address the possibility of impurities, the DMPO was distilled, measured in solution, then tested in a standard Fenton system containing 0.5 mM H_2_O_2_ and 0.1 M Fe^2+^ [42], with the addition of 100 mM DMPO. DMPO/ ⋅ *OH* was observed with relatively minor amounts of impurities, (Fig 3A), suggesting the purity of the DMPO is sufficient for these experiments. Together, these data suggest that the DMPO may not be an effective spin trap in larval and adult zebrafish tissues.

We then assessed the feasibility of detecting $O_{2}^{\cdot -}$ in zebrafish samples using the CMH spin probe. In adult hearts, we observed an oxidized CMH spectrum suggesting the detection of $O_{2}^{\cdot -}$ (Fig 3B). We used a 1 mM concentration of CMH, consistent with established protocols for optimal detection [43].

**Fig 4:**
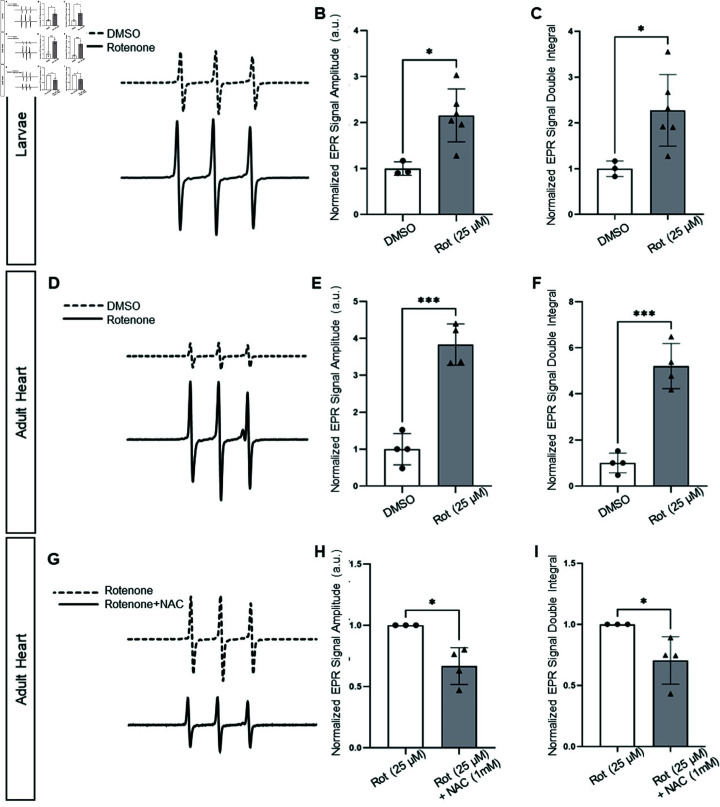
EPR spectra and quantifications of $O_{2}^{\cdot -}$ detected from zebrafish larvae and adult heart tissue using CMH under rotenone and NAC-treated conditions. (A-C) Analysis of $O_{2}^{\cdot -}$ levels in DMSO-treated (control) and rotenone-treated larvae. (D-F) Analysis of $O_{2}^{\cdot -}$ levels in DMSO-treated (control) and rotenone-treated adult hearts. (G-I) Analysis of $O_{2}^{\cdot -}$ levels in rotenone-treated (control) and NAC-treated adult hearts. Representative EPR spectra are shown for controls, DMSO-treated larvae (dashed line; A), DMSO-treated adult hearts (dashed line; D), and rotenone-treated adult hearts (dashed line; G). Representative EPR spectra are shown for rotenone-treated larvae (solid line; A), rotenone-treated adult hearts (solid line; D), and rotenone + NAC-treated adult hearts (solid line; G); quantification of normalized EPR signal amplitude (B, E, H), and normalized double integration (C, F, I). Rotenone treatment increases $O_{2}^{\cdot -}$ levels in larvae (B, C) and adult hearts (E, F) compared to DMSO-treated larvae and adult hearts. NAC reduces $O_{2}^{\cdot -}$ levels in adult hearts following rotenone treatment (H, I). A 1 mM CMH concentration was used for all conditions; n=6 replicates/pools of larvae and n = 4 replicates/adult hearts. NAC, N-acetyl cysteine; Rot, rotenone. Error bar corresponds to SEM.

Next, we increased $O_{2}^{\cdot -}$ production in larvae and adult hearts using rotenone, a specific mitochondrial-derived superoxide radicals inducer which inhibits the complex I of ETC, known to cause oxidative stress (11). By boosting $O_{2}^{\cdot -}$ levels in the samples, we aimed to determine if the CMH probe could reliably detect these increased levels, thus confirming its effectiveness in our experimental setup. As anticipated, significantly higher levels of $O_{2}^{\cdot -}$ species were observed in the rotenone-treated larvae and adult hearts (Fig 4A–4F). The detected amount of $O_{2}^{\cdot -}$ was 2.1-fold higher in rotenone-treated larvae (Fig 4B) and exhibited a 3.8-fold increase in rotenone-treated hearts compared to non-treated samples. Furthermore, we added a commonly used antioxidant drug N-acetyl cysteine (NAC), known for its impact on oxidative stress modulation and effectively reducing production during oxidative stress conditions [44]. We utilized NAC to assess the sensitivity of EPR in quantifying the amount of $O_{2}^{\cdot -}$ reduction in our experimental system, which involved conditions aimed at removing $O_{2}^{\cdot -}$. NAC treatment led to a  ~  1.5-fold reduction in $O_{2}^{\cdot -}$ levels in adult hearts treated with both rotenone and NAC, compared to hearts treated with rotenone alone (Fig 4G–4I), suggesting that acute changes in $O_{2}^{\cdot -}$ levels were detectable by EPR.

To calculate $O_{2}^{\cdot -}$ levels in our samples using CMH, we primarily relied on the signal intensity of each spectrum. However, since the peak amplitude is dependent upon the width, we also measured the second integral, which represents the area under the absorption curve. The results of the double integration closely mirrored the signal intensity of the spectrum peaks, indicating minimal errors in our measurements (Fig 4C, 4F, 4I).

**Fig 5:**
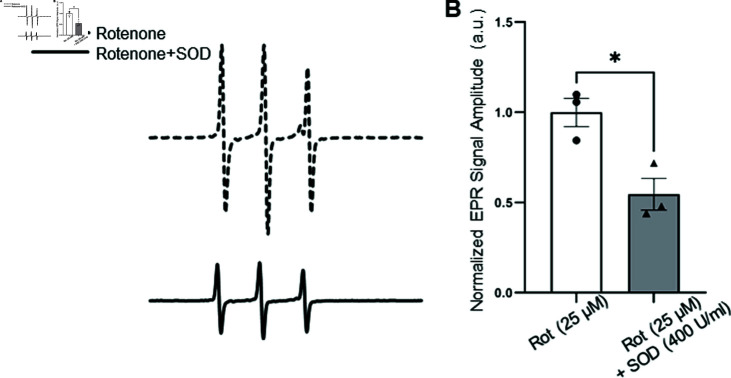
EPR spectra and quantification of $O_{2}^{\cdot -}$ detected from zebrafish adult heart tissue using CMH under rotenone and PEG-SOD treated conditions. Representative EPR spectra are shown for rotenone-treated adult heart (dashed line; A), and rotenone + PEG-SOD treated heart (solid line; A). Quantification of normalized EPR signal amplitude (B). A 1 mM CMH concentration was used for all conditions; n=3 replicates/adult hearts. PEG-SOD, polyethylene glycol-conjugated superoxide dismutase; Rot, rotenone. Error bar corresponds to SEM.

To investigate the contribution of $O_{2}^{\cdot -}$ to the observed EPR signal, cell-permeable polyethylene glycol-conjugated superoxide dismutase (PEG-SOD) was used to catalyze the dismutation of superoxide. PEG-SOD treatment resulted in a 1.83-fold reduction in nitroxide formation compared to hearts treated with rotenone alone (Fig 5A, 5B), confirming that superoxide was the primary contributor to the EPR signal. In addition, a comparison of the signal-to-noise ratios for DMPO in our Fenton reaction system and CMH data in adult hearts (Fig 3) showed values of 172.3 and 889.4, respectively. With scan times of 3.5 minutes for DMPO and 5 seconds for CMH, this suggests that CMH is significantly more sensitive and efficient for detecting $O_{2}^{\cdot -}$. Together, these data suggest that the CMH probe is a highly sensitive and suitable compound for detecting $O_{2}^{\cdot -}$ in larval and zebrafish heart tissue.

**Fig 6:**
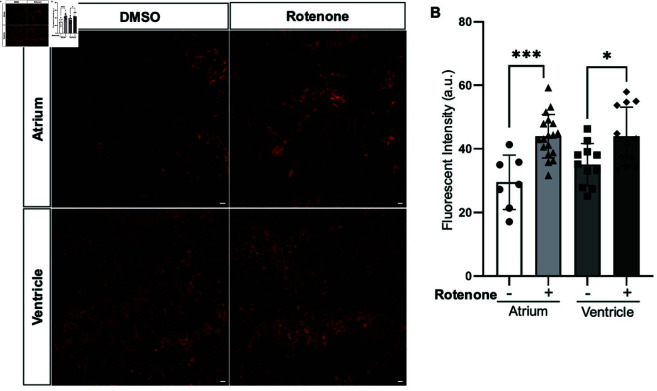
Rotenone-treated heart sections from wild-type adult zebrafish atrium and ventricle indicate an increase in $O_{2}^{\cdot -}$ compared to DMSO-treated samples using DHE. (A) Maximum intensity projections of confocal images from DMSO-treated and rotenone-treated wild-type adult zebrafish atrium and ventricle frozen sections. (B) Quantification of fluorescence intensity from maximum intensity projections reveal a higher amount of $O_{2}^{\cdot -}$ in wild-type adult zebrafish atrium and ventricle sections following rotenone treatment. Scale bar, 10 *μ*m. a.u., arbitrary units. Error bar corresponds to SEM.

To compare the sensitivity of the EPR method in our samples to a common probe-based method, we performed DHE staining on adult zebrafish atria and ventricles that had $O_{2}^{\cdot -}$ levels boosted with rotenone. DHE staining was detected in the non-treated control tissues and rotenone-treated hearts, indicating the presence of $O_{2}^{\cdot -}$ (Fig 6A). Quantification of fluorescence intensity revealed an increase of 43 . 94 ± 1 . 61 compared to 29 . 51 ± 3 . 21 in the atrium ( * *P* < 0 . 0002) and 43 . 9 ± 2 . 65 compared to 35 . 06 ± 1 . 99 ( * *P* < 0 . 01) in the ventricle following rotenone treatment (Fig 6B). The increase in $O_{2}^{\cdot -}$ detected by EPR quantification was 2 . 8-fold higher than in the DHE experiment, suggesting that EPR might offer greater sensitivity for zebrafish samples.

## Discussion

The common approach to studying oxidative stress in zebrafish involves using chemical probes. Some drawbacks associated with the use of such probes include limited sensitivity to low free radical concentrations, issues related to cell permeability, susceptibility to artifact, possible toxic effects, and potential fluorescence background [1,45]. Among different probes, DHE is often employed for detecting $O_{2}^{\cdot -}$. However, previous works showed that using DHE for detecting $O_{2}^{\cdot -}$ has notable drawbacks. Importantly, when DHE reacts with $O_{2}^{\cdot -}$, it primarily produces 2-hydroxyethidium, while ethidium can also be formed, which is not a specific product of the $O_{2}^{\cdot -}$ reaction and can result from non-specific oxidations [46]. Therefore, as ethidium formation reflects broader redox changes rather than specifically indicating $O_{2}^{\cdot -}$, and the fluorescence of these products overlaps, accurate quantification of intracellular $O_{2}^{\cdot -}$ is complicated and can lead to potential inaccuracies [47]. Knowing this limitation, we compared the sensitivity of the EPR method using a CMH probe to the DHE staining technique in our adult zebrafish atria and ventricles. To enhance $O_{2}^{\cdot -}$ production, we treated the samples with rotenone. A lower amount of increase in fluorescence intensity from DHE staining, compared to the quantification detected by EPR, highlights that EPR might offer greater sensitivity. This difference persists even though DHE staining might detect other non-specific radicals besides $O_{2}^{\cdot -}$. Another advanced approach involves the use of genetically encoded redox-sensitive proteins. These engineered redox sensors enable fluorescence-based ratiometric quantification of ROS. Redox sensors offer a great platform for studying oxidative stress but require the development of transgenic animal lines and the use of specialized imaging equipment [11]. The potential use of biosensors in adult tissues raises another issue, which could be addressed with histological approaches. Nevertheless, the risk of artifact oxidation occurring in biosensors during dissection and fixation remains a notable concern [18].

In this study, we detail a precise EPR-based method for detecting and quantifying $O_{2}^{\cdot -}$ in zebrafish larvae and heart tissue. EPR spectroscopy stands out as one of the most accurate methods for the identification of $O_{2}^{\cdot -}$ [43]. EPR relies on fundamental principles of physics and is therefore an essential tool for the direct detection of free radicals in biological systems [33]. While EPR remains a well-established method for studying oxidative stress, defined protocols are limited, particularly for scientists in the fields of biology and biomedical research. Here, we compare the use of DMPO and CMH in zebrafish tissues and identify that the CMH spin probe allows for sensitive and robust detection of $O_{2}^{\cdot -}$. In addition to providing quantitative data on $O_{2}^{\cdot -}$ levels in tissue, EPR may also be valuable in identifying the source of $O_{2}^{\cdot -}$. Previous studies have applied EPR-based techniques to assess NADPH and xanthine oxidase activity [30]. Another notable use of EPR in biological research is to measure mitochondrial semiquinone radicals and Fe-S clusters. This application was demonstrated in a study specifically examining mouse hearts affected by dilated cardiomyopathy [30]. Recent advancements in EPR spectroscopy have enabled the detection of free radicals in live zebrafish, including the identification of melanin radicals and the 5-doxyl stearic acid in zebrafish larvae [48].

One of the primary challenges in EPR is choosing a suitable compound for radical detection. Nitrone spin traps like DMPO form adducts with radicals that yield specific spectra when analyzed using EPR spectroscopy. It is important to note that some spin traps like alpha-phenyl N-tertiary-butyl nitrone (PBN) are more effective for trapping carbon-centered radicals, while others, such as DMPO, are commonly used for trapping $O_{2}^{\cdot -}$ and  ⋅ *OH* radicals [49,50]. In theory, using nitrone spin traps such as DMPO to detect radicals in biological tissues seems ideal. However, in practice, this approach may be less sensitive and complicated by the formation of other radical adducts in complex biological samples. Several considerations should be taken into account in this context. First, nitrone trap radical adducts are unstable, and spin trap reactivity with is relatively low (74 $\text{mol}∕{\text{L}}^{-1}\;{\text{s}}^{-1}$) compared to probes like CMH ($1.2\;\times \;10^{4}$ $\text{mol}∕{\text{L}}^{-1}\;{\text{s}}^{-1}$) [46]. Additionally, they are susceptible to bioreduction in biological samples, making accurate quantification challenging. Another potential issue with DMPO is that after reacting with $O_{2}^{\cdot -}$, original adducts can be rapidly converted to DMPO/ ⋅ *OH* by intracellular glutathione peroxidase within seconds to minutes, potentially making $O_{2}^{\cdot -}$ and hydroxyl radicals indistinguishable [51,52].

In contrast to DMPO, cyclic hydroxylamines like cell-permeable CMH have proven effective for detecting $O_{2}^{\cdot -}$ radicals in tissues [38,46,53]. EPR with cyclic hydroxylamines can also detect site-specific $O_{2}^{\cdot -}$, offering a significant advantage for researchers working with zebrafish, especially given the challenges of applying biosensor lines in the adult stage. Mitochondria-targeted mitoTEMPO-H(1-hydroxy-4-[2-(triphenylphosphonio)-acetamido]-2,2,6,6tetramethylpiperidine) effectively identifies intramitochondrial $O_{2}^{\cdot -}$ in both isolated mitochondria and intact cells and both CMH and mitoTEMPO-H revealed elevated levels of mitochondrial $O_{2}^{\cdot -}$ induced by rotenone [43]. In the present study, we also have found the same effectiveness for CMH to detect $O_{2}^{\cdot -}$ in zebrafish larvae and heart tissue. Unlike spin traps, CMH is oxidized to form stable radicals with a longer half-life, which allows radicals to accumulate and provides quantitative measurements with high sensitivity. EPR using CMH probes is an optimal $O_{2}^{\cdot -}$ detection assay that is sensitive enough to identify any changes, whether an increase or decrease, in the provided system [54]. Furthermore, an important limitation of DMPO lies in its lack of cell permeability, which necessitates tissue lysis prior to EPR analysis. This step not only alters the cellular environment but also introduces a delay between radical formation and detection, during which ROS, being highly unstable, may undergo significant changes [43]. In contrast, cyclic hydroxylamines like CMH are cell-permeable and can be preincubated directly within intact tissue. This advantage allows CMH to access intracellular ROS production sites in a more timely and physiologically relevant manner, reducing the potential for radical alteration [53]. The inherent differences in timing and probe accessibility between DMPO and CMH mean that these methods fundamentally detect ROS under different experimental conditions and cannot be directly compared as parallel approaches.

While our study reports $O_{2}^{\cdot -}$ levels based on signal intensity and double integral measurements, an alternative method to express these data is by normalizing $O_{2}^{\cdot -}$ levels to protein content, using units of pmol/mg protein. This approach offers a standardized measure of $O_{2}^{\cdot -}$ concentration relative to the sample size [43]. Although our current method provides proportional data, expressing results as pmol/mg protein could offer additional clarity and precision for comparisons across different samples. CMH quantification can be accomplished by correlating sample responses with a calibration curve created from known CMH concentrations, which enables accurate measurement of the absolute amount of detected radicals [55]. Another approach for using CMH in animals involves injecting the hydroxylamine probe, then euthanizing the animals and snap-freezing the isolated tissue in a plastic syringe at -80°C. The frozen tissue is later extracted from the syringe and analyzed in a quartz dewar filled with liquid nitrogen to enhance data accuracy and minimize background oxidation [38]. Furthermore, CMH has been successful in detecting intracellular $O_{2}^{\cdot -}$ in cultured cells and tissue samples and exhibits resistance to auto-oxidation. A significant limitation in utilizing CMH probes in EPR assays is their specificity for detecting only $O_{2}^{\cdot -}$. This is attributed to their oxidation process, resulting in the generation of a triple spectrum indicative solely of $O_{2}^{\cdot -}$ presence [36]. It has been shown that supplementation of wild-type mice myocardium samples with PEG-SOD decreased the amount of nitroxide after incubation with CMH, confirming that $O_{2}^{\cdot -}$ is a major contributor to CMH oxidation [56]. In another study, SOD treatment demonstrated that CMH is specific for detecting $O_{2}^{\cdot -}$, as it significantly reduced nitroxide formation, confirming superoxide’s role in CMH oxidation [30]. Similarly, in our study, PEG-SOD treatment decreased nitroxide levels in adult zebrafish hearts treated with rotenone, further demonstrating that $O_{2}^{\cdot -}$ might be the primary target of CMH oxidation.

## Conclusion

EPR proves valuable for investigating oxidative stress in zebrafish larvae and heart tissue. This study introduces a simple, effective, and reliable procedure, enabling the assessment of $O_{2}^{\cdot -}$ in whole larvae and zebrafish hearts. In summary, our use of EPR employing a CMH probe establishes a straightforward assay to quantify oxidative stress in zebrafish.

## Supporting information

S1 Text Step-by-step protocol, also available on protocols.io. (PDF)

S2 Text Source data files (XLSX)
