# Supplementary material for: Electron paramagnetic resonance spectroscopy for analysis of free radicals in zebrafish
Source: PLoS One. 2025 Feb 21;20(2):e0318212. doi: 10.1371/journal.pone.0318212 (PMC11844908; doi:10.1371/journal.pone.0318212)
Supplement: S1 Text — (PDF) [file pone.0318212.s001.pdf]

Electron Paramagnetic Resonance Spectroscopy Protocol for Analysis of Free Radicals in Zebrafish

RESERVED DOI:

10.17504/protocols.io.q26g7mdeqgwz/v1

Mitra Sabetghadam Moghadam[1], Eli Wiens[2], Sébastien Gauvrit[1], Ramaswami Sammynaiken[2], Michelle M. Collins[1]

[1]Department of Anatomy, Physiology, and Pharmacology, College of Medicine, University of Saskatchewan, Saskatoon, SK, Canada;

[2]Saskatchewan Structural Sciences Centre, University of Saskatchewan, Saskatoon, SK, Canada

Michelle Collins

University of Saskatchewan

Protocol Info: Mitra Sabetghadam Moghadam, Eli Wiens, Sébastien Gauvrit, Ramaswami Sammynaiken, Michelle M. Collins . Electron Paramagnetic Resonance Spectroscopy Protocol for Analysis of Free Radicals in Zebrafish. **protocols.io**
https://protocols.io/view/electron-paramagnetic-resonance-spectroscopy-proto-dxgx7jxn

Created: January 06, 2025

Last Modified: January 08, 2025

Protocol Integer ID: 118007

Keywords: Zebrafish, Electron paramagnetic spectroscopy, Spin trap, Spin probe

Funders Acknowledgements:
National Sciences and Engineering Research Council of Canada Discovery Grant
Grant ID: RPGIN-2022-04756
Saskatchewan Health Research Fund
Grant ID: Establishment Grant
University of Saskatchewan College of Medicine
Heart and Stroke Foundation of Canada
Grant ID: New Investigator Award

Abstract

This protocol details the analysis of free radicals in zebrafish by electron paramagnetic resonance spectroscopy method.

Guidelines

INTRODUCTION

There are two key approaches in EPR spectroscopy: using spin traps and spin probes. EPR can be performed using a chemical spin trap that reacts with biological samples containing unpaired electrons to create a spin adduct. Spin traps produce specific spin adducts depending on the type of original free radical, resulting in specific spectra **[1]**. 5,5-dimethyl-1-pyrroline-N-oxide (DMPO) is a widely used nitrone spin trap capable of reacting with distinct free radicals **[2]**, such as $O_2^{\cdot-}$ and ●OH, generating DMPO/●OOH and DMPO/●OH adducts, respectively. These adducts are then captured using the EPR spectrometer as a specific spectrum. The use of spin probes for detecting ROS in biological samples has been widely employed. One class of compounds, the cyclic hydroxylamines, has proven extremely effective for use in tissues and cultured cells. Probes do not react with free radicals to form a covalent bond. Instead, probes are oxidized by free radicals present in the system, forming an oxidized form of the probe, nitroxide, with a half-life of several hours which is detectable by EPR **[3]**. The cyclic hydroxylamine probe, 1-hydroxy-3-methoxycarbonyl-2,2,5,5-tetramethylpyrrolidine (CMH), is oxidized and produces CM• nitroxide in the presence of $O_2^{\cdot-}$.

EPR has been used for detecting free radicals such as ROS in various biological samples, including frozen biopsies **[4]**, blood **[5]**, and animal models including zebrafish embryos **[6]**, mice **[7]**, and pigs **[8]**. Here, we compare the use of DMPO spin trap and CMH spin probe and present an optimized EPR-based method to measure the most abundant ROS, superoxide ($O_2^{\cdot-}$) in whole larvae and isolated hearts from juvenile and adult zebrafish.

Materials

Materials:

- **DMPO Spin Trap EPR**

1. 5,5-Dimethyl-1-pyrroline-N-oxide **Enzo Life Sciences Catalog #**3317-61-1
2. Sodium Orthovanadate **Merck MilliporeSigma (Sigma-Aldrich) Catalog #**S6508-10G
3. Sodium Chloride **Merck MilliporeSigma (Sigma-Aldrich) Catalog #**S7653
4. β-Glycerophosphate disodium salt hydrate **Merck MilliporeSigma (Sigma-Aldrich) Catalog #**G9422
5. Sodium pyrophosphate dibasic **Merck MilliporeSigma (Sigma-Aldrich) Catalog #**P8135
6. Ethylenediaminetetraacetic acid, disodium salt dihydrate (EDTA) (ThermoFisher Scientific, catalog number: 409971000)
7. Ethylene glycol-bis(2-aminoethylether)-N,N,N′,N′-tetraacetic acid (25g) **Merck MilliporeSigma (Sigma-Aldrich) Catalog #**E3889
8. Triton™ X-100, 98%, for molecular biology, DNAse, RNAse and Protease free **Thermo Fisher Scientific Catalog #**327372500

   TRIS hydrochloride **Merck MilliporeSigma (Sigma-Aldrich) Catalog #**PHG0002
9. Pierce Protease Inhibitor Tablets **Thermo Fisher Catalog #**A32963

- **CMH Spin Probe EPR**

1. 1-Hydroxy-3-methoxycarbonyl-2,2,5,5-tetramethylpyrrolidine **Enzo Life Sciences Catalog #**ALX-430-117-M010
2. Deferoxamine mesylate salt **Merck MilliporeSigma (Sigma-Aldrich) Catalog #**D0160000
3. Sodium diethyldithiocarbamate trihydrate **Merck MilliporeSigma (Sigma-Aldrich) Catalog #**228680
4. Krebs-Ringer solution, HEPES-buffered (ThermoFisher Scientific, catalog number: J67795.AP)

- **DHE Staining**

1. Dihydroethidium (DHE) (Sigma-Aldrich, product number: S6792)
2. Tissue-Plus™ O.C.T. Compound **Fisher Scientific Catalog #**23-730-571

- **General Materials**

1. Rotenone **Merck MilliporeSigma (Sigma-Aldrich) Catalog #**R8875
2. N-Acetyl-L-cysteine **Merck MilliporeSigma (Sigma-Aldrich) Catalog #**A9165
3. Superoxide dismutase–polyethylene glycol **Merck MilliporeSigma (Sigma-Aldrich) Catalog #**S9549
4. DMEM/F-12, GlutaMAX™ supplement **Thermo Fisher Catalog #**10565018
5. Dimethyl Sulfoxide, Fisher BioReagents™ **Fisher Scientific Catalog #**BP231-1
6. Phosphate-buffered saline (PBS): 137 mM NaCl, 2.7 mM KCl, 10 mM Na2HPO4, 1.8 mM KH2PO4 in deionized H2O, adjust the pH to 7.4 with HCl, autoclave, and store at room temperature.
7. Sea salt (Instant Ocean)
8. Ethyl 3-aminobenzoate methanesulfonate **Merck MilliporeSigma (Sigma-Aldrich) Catalog #**E10521

Equipment:

- Surgical tools: sterile scalpels (ThermoFisher Scientific, catalog number: 12460454), straight forceps (F.S.T, item number. 11255-20, Dumont #55)

Equipment	
**Sterile Standard Scalpels**	NAME
Scalpels	TYPE
Integra™ Miltex	BRAND
12-460-454	SKU
https://www.fishersci.com/shop/products/integra-miltex-sterile-standard-scalpels-7/12460454	LINK

Equipment	
**Straight Forceps**	NAME
Forceps	TYPE
Fine science tools	BRAND
11255-20 Dumont #55	SKU
https://www.finescience.com/en-US/Products/Forceps-Hemostats/Dumont-Forceps/Dumont-55-Forceps/11255-20	LINK

- 1.4 mm ceramic beads (Cole-Parmer, 19-645-3)
- Homogenizer (OMNI Bead-Mill)
- Tabletop centrifuge
- 15 ml Falcon tubes (ThermoFisher Scientific, catalog number: 14-959-49B)

Equipment

**15 mL Conical Centrifuge Tubes**	NAME
Falcon tubes	TYPE
Fisher	BRAND
14-959-49B	SKU
https://www.fishersci.com/shop/products/falcon-15ml-conical-centrifuge-tubes-5/1495949B	LINK

- 1.5 ml Eppendorf tubes
- Microscope slides (ThermoFisher Scientific, catalog number: 1255015)

Equipment

**Superfrost™ Plus Microscope Slides**	NAME
Slides	TYPE
Fisherbrand™	BRAND
12-550-15	SKU
https://www.fishersci.com/shop/products/fisherbrand-superfrost-plus-microscope-slides-2/1255015	LINK

- Glass coverslips (ThermoFisher Scientific, catalog number: 12-548-B)

Equipment	
**Cover Glasses**	NAME
Coverslips	TYPE
Fisher	BRAND
12-548B	SKU
https://www.fishersci.com/shop/products/fisherfinest-premium-cover-glasses-14/12548B#?keyword=12-548-B	LINK

- Wilmad® quartz (CFQ) EPR tubes; O.D. 4 mm, L 100 mm (Sigma-Aldrich, product number: Z567361)

Equipment	
**Wilmad® quartz (CFQ) EPR tubes**	NAME
EPR tubes	TYPE
Merck	BRAND
Z567361	SKU
https://www.sigmaaldrich.com/IN/en/product/aldrich/z567361?srsltid=AfmBOooWW0UBSFCcTV1sZ75w-QCpnE2nCSCacEf0M-VCkldEdAT3WfOl	LINK

- Glass capillary tubes PYREX® 90 mm Capillary Melting Point Tubes, One End Open (Product number: 9530-3)
- Liquid nitrogen flask (ThermoFisher Scientific)
- Brüker EMX EPR with ER 4119 HS cavity
- Xenon software (version 1.1b60)
- CryoStar NX50 cryostat (Thermo Scientific)
- ImageJ software

Before start

Before starting this protocol, ensure that animal ethics are in place and confirm your Institution’s approved animal euthanasia protocols. Sample sizes are described below. Statistical analyses of EPR data may be performed using unpaired t-tests using GraphPad Prism. Groups of data are significantly different at a p-value <0.05.

DMPO Spin Trap EPR

1h 2m

1 **Reagent Setup**

1.1 Prepare a [M] 200 millimolar (mM) stock solution of sodium orthovanadate by dissolving 1.84 g in 50 mL double-distilled water. Aliquot and store at -20 °C . Solution remains stable for at least one year at this temperature. After defrosting, vortex to dissolve any crystals.

1.2 Prepare a [M] 200 millimolar (mM) stock solution of β-glycerophosphate disodium salt hydrate by dissolving 2.16 g in 50 mL of double-distilled water. Aliquot and store at -20 °C . Ensure the storage container is sealed to protect it from moisture and light.

1.3 Prepare [M] 200 millimolar (mM) stock solution of sodium pyrophosphate dibasic by dissolving 4.44 g in 100 mL double-distilled water. Mix the solution thoroughly to ensure complete combination. Using a magnetic stirrer and microwaving for one minute can facilitate the dissolving process. Store at Room temperature .

1.4 Prepare a [M] 1 Molarity (M) DMPO stock solution by weighing 163.2 mg of DMPO and dissolving it in 1 mL of distilled water.

1.5 Prepare lysis buffer with protease inhibitor mixture (29).
Lysis buffer:

A	B
Tris-HCl, pH 7.4	20 mM
Sodium orthovanadate	1 mM
β-glycerophosphate disodium salt hydrate	1 mM
EGTA	1 mM
Sodium pyrophosphate dibasic	2.5 mM
Sodium chloride	150 mM
EDTA	1 mM
Triton X-100	1%

2 **Sample preparation: zebrafish larvae**

2.1 Raise zebrafish in egg water at 28 °C until the desired stage is reached.

2.2 Anesthetize larvae using buffered tricaine at 0.015% in egg water.

2.3 Transfer 20 larvae to a 1.5 ml tube and remove as much egg water as possible.

2.4 Add 100 µL lysis buffer.

2.5 Incubate On ice for 00:10:00 - 00:15:00 .

15m

2.6 Centrifuge at 12000 rpm, 4°C, 00:15:00 .

15m

2.7 Transfer the supernatant (protein lysate) to a new 1.5 ml tube. immediately.

3 **Sample preparation: adult zebrafish heart**

3.1 Euthanize adult fish according to approved animal welfare methods (i.e., hypothermia followed by decapitation).

3.2 Dissect the heart from the fish. The heart is positioned posterior and ventral to the gill. Cut out the heart and be careful to avoid damaging the atrium.

3.3 Wash the heart in a petri dish with 1X PBS.

3.4 Place the heart immediately in DMEM medium. You may still observe the heart beating.

3.5 Transfer and combine three hearts in a 2 ml tube.

3.6 Add 100 µL of lysis buffer and ~10 beads. Then, homogenize hearts for 00:02:00 at a speed of 4 m/s using a homogenizer.

2m

3.7 Incubate homogenate On ice for 00:10:00 - 00:15:00 . 15m

3.8 Centrifuge at 12000 rpm, 4°C, 00:15:00 . 15m

3.9 Transfer supernatant to a new 1.5 ml. Begin step 4 immediately.

4 **EPR measurement and software analysis**

4.1 Add 10 µL of the [M] 1 Molarity (M) DMPO stock solution to the lysate to make a total volume of 100 µL with a final concentration of [M] 100 millimolar (mM) DMPO immediately after sample preparation.

4.2 Transfer the aqueous solution containing homogenate and DMPO to capillary tubes and place it in the cavity of the EPR machine.

4.3 Measure the EPR spectra using the following parameters: scan width: 125 G, spectral resolution: 0.1 G, scan time: 30 s, averaged scans: 4, microwave power 6 mW, microwave frequency ~9.86 GHz, modulation frequency 100 kHz, modulation amplitude 1.0 G.

4.4 Simulate EPR spectra with the Xenon processing software using SpinFit. Enter the parameters of the identified radicals and fit the peak areas. Show the residual to look for any components that were missed during the set-up of the simulation.

CMH Spin Probe EPR

3h 20m

5 **Reagent Setup**

5.1 Prepare a [M] 10 millimolar (mM) stock solution of CMH by dissolving 10 mg in 3 mL of double-distilled water. Aliquot the solution into dark glass containers to avoid multiple freeze-thaw cycles and store it at -20 °C .

- Rapidly freeze the aliquots immediately after preparation to minimize exposure to oxygen. Although CMH is ideally stored under an inert atmosphere to further reduce autooxidation, we ensured the integrity of the probe by using it immediately after defrosting for each experiment.
- To verify stability, Routinely perform EPR measurements on CMH solutions without samples, confirming no significant autooxidation or background signal under these

conditions.

5.2 Prepare a [M] 70 millimolar (mM) stock solution of deferoxamine mesylate salt by dissolving 0.5 g in 10 mL of double-distilled water. Store at -20 °C for several weeks.

5.3 Prepare a [M] 500 millimolar (mM) stock solution of sodium diethyldithiocarbamate trihydrate by dissolving 1 g in 10 mL of double-distilled water. Store at -20 °C for several weeks.

5.4 Prepare a fresh [M] 1 millimolar (mM) CMH solution in Krebs-Hepes buffer (KHB) with [M] 5 micromolar (µM) sodium diethyldithiocarbamate trihydrate and [M] 25 micromolar (µM) deferoxamine methane-sulfonate salt. Place CMH solution On ice and protect it from the light.

6 **Sample preparation: zebrafish larvae**

6.1 Anesthetize larvae using buffered tricaine at 0.015% in egg water.

6.2 Transfer 20 larvae to a 1.5 ml tube and remove as much egg water as possible.

6.3 before adding the CMH solution if you want to treat samples with antioxidants (e.g., N-acetyl cysteine) or an oxidant solution (e.g., rotenone).

6.4 Add 50 µL CMH solution per 20 larvae. immediately.

7 **Sample preparation: adult zebrafish heart**

7.1 Euthanize adult fish according to approved animal welfare methods (i.e., hypothermia followed by decapitation).

7.2 Dissect the heart from the fish. The heart is positioned posterior and ventral to the gill. Cut out the heart and be careful to avoid damaging the atrium.

7.3 Wash the heart in a petri dish with 1X PBS.

7.4 Remove the bulbus arteriosus from the ventricle and separate the chambers (if necessary).

7.5 Place the heart or isolated chambers immediately in DMEM medium. If done quickly, you may still observe the heartbeat.

7.6 Transfer and combine three hearts in a 2 ml tube. before adding the CMH solution if you want to treat samples with antioxidants (e.g., N-acetyl cysteine) or an oxidant solution (e.g., rotenone).

7.7 Add 50 µL CMH solution per three hearts. immediately.

8 Rotenone and NAC/PEG-SOD treatment

8.1 **Larval treatment:** 1h

1. Place 20 larvae in egg water containing DMSO rotenone ([M] 25 micromolar (µM)), or rotenone ([M] 25 micromolar (µM)) and NAC ([M] 1 millimolar (mM)).
2. Incubate for 01:00:00 at 28 °C .
3. Remove treatment solutions and add 50 µL CMH solution per 20 larvae. immediately.

8.2 **Adult heart tissue:** 1h 20m

1. Place three hearts immediately in DMEM medium containing DMSO, rotenone ([M] 25 micromolar (µM)), or rotenone ([M] 25 micromolar (µM)) and NAC ([M] 1 millimolar (mM)) or rotenone ([M] 25 micromolar (µM)) and PEG-SOD (400 U/ml).
2. Incubate the NAC+rotenone samples for 01:00:00 at 28 °C and the PEG-SOD treatment samples for 00:15:00 - 00:20:00 at 37 °C . Remove treatment solutions and add 50 µL CMH solution per three hearts. immediately.

9 CMH Incubation

9.1 Incubate samples in CMH for 01:00:00 at 37 °C . 1h

9.2 Remove the entire volume of CMH solution from the tubes after 1 hr.

9.3 Freeze samples in liquid nitrogen. This quick-freezing method aids in keeping samples fresh for storage or analysis at a later time, and it makes handling and transferring samples to EPR

tubes easier, especially for small sample sizes. Freezing might not be required if you want to measure samples immediately after incubation.

10 **EPR measurement and software analysis**

10.1 Transfer the frozen samples to the center of the EPR tube. Keep in mind that the sample position can change the signal intensity, therefore consistent sample positioning ensures uniform signal intensity.

10.2 Place EPR tubes containing samples in the cavity of the EPR machine.

10.3 Open the Bruker Xenon software and record EPR spectra following the instructions outlined in the Xenon user guide documentation, according to the recommended settings: the microwave power, 20-21.9 mW; magnetic field center 3,386 G; modulation frequency, 86-100 kHz; modulation amplitude, 0.5-2.5 G; time constant, 82 ms; scan time, 41 s; and number of scans 1.

10.4 Utilize the Bruker Xenon software to measure the intensity of detected peak(s) and perform double integrals of signals, following the guidelines outlined in the provided Xenon user guide documentation.

11 **Key Considerations for EPR Analysis of Juvenile Zebrafish Hearts**

- We also conducted EPR using a CMH probe on hearts isolated from juvenile zebrafish. The sample preparation followed the same protocol as for adult zebrafish hearts, with the main difference being that we used five juvenile hearts instead of three because of their smaller size.
- Handling and transferring juvenile hearts to EPR tubes can be challenging due to their sizes.
- To address this, we recommend placing the hearts at the top of a glass capillary tube (which can be done under a brightfield microscope) and then inverting the capillary tube into an EPR tube. It's crucial to position the EPR tube correctly within the EPR machine's cavity for accurate measurements.

DHE Staining

1h 15m

12 **Sample preparation: adult zebrafish heart**

12.1 Euthanize adult fish according to approved animal welfare methods (i.e., hypothermia followed by decapitation).

12.2 Dissect the heart from the fish. The heart is positioned posterior and ventral to the gill. Cut out the heart and be careful to avoid damaging the atrium.

12.3 Wash the heart in a petri dish with 1X PBS.

12.4 Remove the bulbus and separate chambers, if needed.

13 **DHE staining**

13.1 Place the heart immediately in DMEM medium containing DMSO or rotenone ([M] 25 micromolar (µM)).

13.2 Incubate for 01:00:00 at 28 °C . 1h

13.3 Remove the treatment solution and rinse hearts in ice-cold PBS buffer.

13.4 Embed hearts in OCT compound in a mold/cassette.

13.5 Place samples immediately at -80 °C to freeze OCT.

13.6 Section at 10 µm thickness using a cryostat.

13.7 Remove OCT by briefly washing slides with water.

13.8 Incubate slides for 00:15:00 in DHE ([M] 5 micromolar (µM)) at Room temperature . 15m

13.9 Wash twice with deionized water

13.10 Add coverslips to the slide.

13.11 Image using a confocal microscope at 640 nm.

Protocol references

References:

1. Gotham JP, Li R, Tipple TE, Lancaster JR, Jr., Liu T, Li Q. Quantitation of spin probe-detectable oxidants in cells using electron paramagnetic resonance spectroscopy: To probe or to trap? Free radical biology & medicine. 2020;154:84-94. Epub 2020/05/08. doi: 10.1016/j.freeradbiomed.2020.04.020. PubMed PMID: 32376456; PubMed Central PMCID: PMCPMC7368495.
2. Zhang H, Joseph J, Vasquez-Vivar J, Karoui H, Nsanzumuhire C, Martasek P, et al. Detection of superoxide anion using an isotopically labeled nitrone spin trap: potential biological applications. FEBS Lett. 2000;473(1):58-62. Epub 2000/05/10. doi: 10.1016/s0014-5793(00)01498-8. PubMed PMID: 10802059.
3. Dikalov SI, Polienko YF, Kirilyuk I. Electron Paramagnetic Resonance Measurements of Reactive Oxygen Species by Cyclic Hydroxylamine Spin Probes. Antioxid Redox Signal. 2018;28(15):1433-43. Epub 2017/10/19. doi: 10.1089/ars.2017.7396. PubMed PMID: 29037084; PubMed Central PMCID: PMCPMC5910043.
4. Berg K, Ericsson M, Lindgren M, Gustafsson H. A high precision method for quantitative measurements of reactive oxygen species in frozen biopsies. PloS one. 2014;9(3):e90964. Epub 2014/03/08. doi: 10.1371/journal.pone.0090964. PubMed PMID: 24603936; PubMed Central PMCID: PMCPMC3947958.
5. Mrakic-Sposta S, Gussoni M, Montorsi M, Porcelli S, Vezzoli A. Assessment of a standardized ROS production profile in humans by electron paramagnetic resonance. Oxid Med Cell Longev. 2012;2012:973927. Epub 2012/08/18. doi: 10.1155/2012/973927. PubMed PMID: 22900129; PubMed Central PMCID: PMCPMC3412105.
6. Zhang Y, Shimizu H, Siu KL, Mahajan A, Chen JN, Cai H. NADPH oxidase 4 induces cardiac arrhythmic phenotype in zebrafish. The Journal of biological chemistry. 2014;289(33):23200-8. Epub 2014/06/26. doi: 10.1074/jbc.M114.587196. PubMed PMID: 24962575; PubMed Central PMCID: PMCPMC4132817.
7. Lynch TLt, Sivaguru M, Velayutham M, Cardounel AJ, Michels M, Barefield D, et al. Oxidative Stress in Dilated Cardiomyopathy Caused by MYBPC3 Mutation. Oxid Med Cell Longev. 2015;2015:424751. Epub 2015/10/29. doi: 10.1155/2015/424751. PubMed PMID: 26508994; PubMed Central PMCID: PMCPMC4609873.
8. Dudley SC, Jr., Hoch NE, McCann LA, Honeycutt C, Diamandopoulos L, Fukai T, et al. Atrial fibrillation increases production of superoxide by the left atrium and left atrial appendage: role of the NADPH and xanthine oxidases. Circulation. 2005;112(9):1266-73. Epub 2005/09/01. doi: 10.1161/CIRCULATIONAHA.105.538108. PubMed PMID: 16129811.
